# “What Do We Do Now?”: Recommendations to Combat Professionals’ Public Stigma Against Women Who Experience Intimate Partner Violence

**DOI:** 10.1007/s10508-025-03156-9

**Published:** 2025-07-07

**Authors:** Lara Murvartian, Francisco Javier Saavedra-Macías

**Affiliations:** 1 Faculty of Psychology, University of Seville, Camilo José Cela, s/n, 41018 Seville, Spain; 2 Faculty of Psychology, University of Seville, Seville, Spain

**Keywords:** Public stigma, Intimate partner violence against women, Integrated care, Health

## Abstract

Professionals’ public stigma toward intimate partner violence against women (IPVAW) survivors represents a significant barrier to the recovery from violence. The aim was to analyze the actions that professionals working in various sectors involved in integrated care consider relevant to preventing professionals’ stigma. Semistructured in-depth interviews were conducted with 25 professionals working in Spain. A thematic analysis was employed. The recommendations suggested by the participants were divided into two main themes: actions to prevent stigma in society and actions to prevent stigma among professionals. The latter were further divided into individual-level actions and structural-level actions. Among the actions to prevent stigma in society, emphasis was placed on promoting gender equality in a cross-cutting manner. At the structural level, some of the suggestions were to provide institutions with resources and protocols to provide IPVAW-specialized services and to ensure competent professionals through effective training and community workspaces to facilitate group discussions and supervision, among other actions. Additionally, evaluating the real effectiveness of psychosocial intervention programs targeting survivors was suggested. At the individual level, professionals should engage in self-reflection regarding their own stigma and refer to other colleagues when necessary. A survivor-centered intervention model was also advocated, which seeks to empower women and is based on the strengths of survivors and healthy relationships. This also implied that social intervention professionals should address IPVAW before considering the removal of minors. This study offers relevant recommendations to combat the stigma at all levels, contributing to high-quality professional care for survivors.

## Introduction

Intimate partner violence against women (IPVAW) is a public challenge (Dobash & Dobash, 1979; United Nations, 2023; World Health Organization [WHO], 2021) that continues to be the most common form of violence against women. Almost 1 in 3 ever partnered women worldwide have reported experiencing physical and/or sexual IPVAW at least once in their lifetime (WHO, 2021). IPVAW includes controlling behavior, psychological abuse, physical aggression, and/or sexual coercion by an intimate (ex)partner (WHO, 2021). It is a public health issue with serious short-, medium- and long-term psychological, physical and/or reproductive consequences for women survivors, which extend beyond the termination of the relationship, and some of which are fatal (García Moreno et al., 2005; WHO, 2021). It also has financial and social costs for survivors (WHO, 2021) and serious repercussions for the health of minors and other dependents (García-Moreno et al., 2005; WHO, 2013).

The recognition of IPVAW as a public problem (United Nations, 2023; WHO 2021) has led to an increased focus on social and community-based factors in efforts to prevent and combat IPVAW (Ferrer-Pérez et al., 2019; WHO, 2019). Among these factors, there has been a recent focus on the acceptance of false beliefs and negative attitudes toward IPVAW (Ferrer-Pérez et al., 2019; Gracia et al., 2020). These factors contribute to a negative social response toward survivors, even among professionals who are responsible for providing support services (Serrano-Montilla et al., 2023). Social and community-based factors also include public stigma toward women survivors of IPVAW (Murvartian et al., 2023b, 2024a), a complex concept that includes false beliefs, negative attitudes and responses that has been less frequently addressed in the literature. The present study focuses on recommendations to prevent the public stigma exerted by professionals toward IPVAW survivors. Next, we will explain the nature of this stigma.

### Public Stigma Toward Survivors of Intimate Partner Violence Against Women

Public stigma is a process that emerges in social relations. First, certain differences that contradict social norms about what is considered acceptable, desirable or normal (e.g., profession, physical appearance) are labeled. This results in a separation between the people who are categorized under that label and those who are not. As a result of categorization, the variability between members of the same group is ignored, and they are reduced to the assigned label. Second, depending on the prevailing social values, certain negative and stereotyped beliefs are associated with the stigmatized social group (Goffman, 1963; Link & Phelan, 2001), which results in a reduction in social status (Link & Phelan, 2001). Stigma is therefore shaped by the sociocultural and historical context in which it emerges (Goffman, 1963; Pescosólido & Martín, 2015). If such negative beliefs are accepted, they can lead to discriminatory behaviors, which in turn reinforce and maintain negative beliefs.

Overstreet and Quinn (2013) were pioneers in applying stigma theory to IPVAW. They found that the label “IPVAW survivor” was associated with a stereotype of women being weak, passive, and responsible for abuse. This, in turn, led to isolation, blaming, and judgmental behaviors by third parties.

Regarding stigma perpetuated by professionals, the literature has identified several factors that constitute the roots of this stigma. In the literature, some of the most prevalent are patriarchal social norms and a lack of knowledge on IPVAW dynamics and recovery (Murray et al., 2016; Murvartian et al., 2023a, 2023b, 2024a, 2024b). Other factors include personal experiences with IPVAW (Murvartian et al., 2023a, 2024b), survivors or perpetrators, and privileged identities (e.g., being a man, heterosexual, etc.) that increase the power imbalance between professionals and survivors (Murray & Crowe, 2017; Murvartian et al., 2023a, 2024b). Additionally, the symptom-focused approach of healthcare professionals can obscure the underlying cause of symptoms—namely, IPVAW—as well as the unique circumstances and needs of each survivor. It is essential to understand the roots of this stigma to develop effective strategies to combat them.

According to recent literature, the aforementioned factors are associated with erroneous beliefs about IPVAW and survivors that professionals have internalized, such as the erroneous idea that there is a survivor profile (Murray et al., 2016; Murvartian et al., 2023a, 2023b, 2024a, 2024b; Overstreet & Quinn, 2013). In addition, the roots of stigma are associated with stigmatizing behaviors that occur in professional care, which in turn are underpinned by the aforementioned erroneous beliefs. This behavioral component reinforces erroneous beliefs. Examples of stigmatizing behaviors include blaming survivors of abuse or intervening without hope that they will recover from IPVAW (Murvartian et al., 2023a, 2024b).

Because this study focuses on professional practice, it is worth highlighting that patriarchal beliefs shape not only public discourse but also professional environments, influencing how IPVAW survivors are perceived and treated. Workplaces, much like any other sites, such as the media, political institutions, or educational settings, serve as sites where gender biases and exclusionary practices are reproduced (Acker, 1990; Connell, 2009). According to Acker (2006), inequalities within work organizations are maintained through “inequality regimes,” a set of interwoven practices and processes that perpetuate disparities in power, opportunities, and treatment based on gender, class, and race, among other stigmatized social identities. These regimes shape workplace norms and reinforce hierarchical structures that contribute to the minimization of IPVAW, the silencing of survivors, and the perpetuation of stigma (Murvartian et al., 2023a, 2023b, 2024a, 2024b). Such institutionalized inequalities intersect with broader societal narratives, reinforcing professionals’ biases toward survivors. This highlights the need for systemic interventions that challenge not only individual attitudes but also structural barriers that sustain gendered stigma.

Finally, it is worth adding that one of the challenges in eliminating public stigma is the subtle and implicit nature of stigmatizing myths and behaviors. In most cases, public stigma is manifested through gestures, silences, etc., that indicate excessive friendliness, doubt, superiority, etc. These are perceived by survivors (Overstreet & Quinn, 2013; Woods, 2010) and are difficult for professionals to identify and self-monitor (Link & Phelan, 2001).

### Consequences of Professionals’ Stigma

The WHO has highlighted the importance of health professionals, as they are responsible for providing the clinical treatment needed by survivors and act as a key gateway for the early detection of IPVAW, especially when there is no disclosure of abuse (Garcia-Moreno et al., 2005; Rizkalla et al., 2020). They also facilitate information and contact with other support services. Furthermore, individuals who have experienced traumatic events are more likely to utilize primary healthcare services (Woods, 2010). However, the WHO (2019) emphasizes the necessity for an integrated, multidisciplinary response involving professionals from various sectors (law enforcement, legal, health, psychological and social intervention fields).

The consequences of stigma from professionals are severe. First, when survivors are aware of stigma (sometimes because they have experienced it before), they may anticipate it and be deterred from disclosing the abuse and/or seeking help. Moreover, they may end up internalizing the stigma they have encountered, making it difficult to redisclose the abuse and/or seek help again (Durán-Martín et al., 2022; Overstreet & Quinn, 2013). Another cost of stigma is the lack of detection of IPVAW, which has a detrimental impact on women’s health and may even place their lives at risk (Murvartian et al., 2024b).

Stigmatizing survivors can lead to feelings of incomprehension and helplessness, as well as an overexertion in proving that they are acting “correctly” in the eyes of professionals. This may result in burnout, which can sometimes progress to depressive symptoms or posttraumatic stress disorder, among other consequences (Kennedy & Prock, 2018; Meyer, 2016). Ultimately, survivors may choose to cease involvement with support services. Furthermore, stigma increases the likelihood that women will remain in abusive relationships and exacerbates the challenges they already face during recovery from IPVAW (e.g., difficulties in finding employment; Tarshis, 2020).

A key aspect in understanding the functioning of public stigma is that it arises in a context in which there is a power imbalance between the person who stigmatizes and the stigmatized. Power that is exercised at the macro level (through social norms, legislation, etc.) is transferred to interpersonal relationships (Foucault, 1976). Thus, an individual’s stigmatizing beliefs and behaviors reflect the patriarchal sociocultural context that defines what is considered acceptable regarding the use of violence against women in intimate relationships (Gracia et al., 2020; Overstreet & Quinn, 2013). In addition, it is necessary to understand that public stigma is a mechanism of social control over the stigmatized group (Link & Phelan, 2001). In this case, the stigmatization of survivors reinforces gender inequalities inherent in the patriarchal system, which vary across different social contexts. Stigma contributes to men continuing to hold power over resources and life opportunities over women (Barnett et al., 2016; Dobash & Dobash, 1979).

However, this power imbalance does not affect all survivors equally. The impact of stigma is shaped by intersecting systems of oppression, meaning that those who hold multiple marginalized identities, such as being Black or an immigrant, face compounded barriers when seeking support (Crenshaw, 1991; Huelley et al., 2023). For instance, negative stereotypes about Black women influence how their experiences of violence are perceived and addressed (Crenshaw, 1991). Recent literature continues to highlight the prevalence of social rejection and racism (Waller et al., 2022). Moreover, the anti-violence against women movement has not always prioritized Black women’s perspectives, limiting a comprehensive understanding of their needs. Similarly, mainstream feminist discussions have often overlooked their distinct realities, shaped by prevailing stereotypes. As a result, Black women survivors may hesitate to seek support services, fearing the reinforcement of negative stereotypes or a lack of understanding of their specific circumstances. For example, the harmful stereotype portraying Black women as inherently aggressive can lead them to doubt whether they will be recognized as victims, potentially fearing suspicion or blame when seeking assistance (Crenshaw, 1991).

Immigrant survivors also encounter additional barriers when accessing support. These challenges may be linked to their immigration status, which can trap them in abusive situations due to fear of deportation. Furthermore, support services are not always adequately equipped to provide culturally sensitive or linguistically appropriate assistance, further disadvantaging immigrant survivors (Crenshaw, 1991; Huelley et al., 2023).

When these power asymmetries extend to the provider-survivor relationship, they heighten the risk of discrimination and status loss (2023b; Murvartian et al., 2023a, 2024a, 2024b; Barrios et al., 2021). This dynamic not only exacerbates existing inequalities but also limits survivors’ willingness to seek help, ultimately hindering their recovery process (Crenshaw, 1991; Huelley et al., 2023; Murray et al., 2016). It is vital to prevent stigma from professionals (Durán-Martínez et al., 2022; WHO, 2021) and to promote informed care that is respectful of the moment of recovery and based on active listening and compassion (Cubells & Calsamiglia, 2018; García-Moreno et al., 2005; Murray et al., 2016). It is also important to consider the cultural context (Murray & Crowe, 2017; Sakellari et al., 2024) and the individual needs of women (Murray et al., 2016; Smye et al., 2020). In this regard, an intersectional approach is essential, as it enables the recognition and proper response to the unique challenges faced by marginalized survivors (Murvartian et al., 2024b). Finally, all of these factors point to the importance of involving survivors in decision-making to ensure care that is truly responsive to their needs (Durán-Martínez et al., 2022; WHO, 2021).

### Efforts to Combat this Stigma: Achievements and Challenges

A growing body of literature has shown that professionals who receive training in violence against women (VAW) and in how to adequately assist survivors provide better care (e.g., Almqvist et al., 2018; Sakellari et al., 2024). However, is training currently effective in reducing stigma? A comprehensive literature review was conducted (Murvartian et al., 2023b, 2024a) to identify actions to reduce professionals’ public stigma against VAW survivors. To our knowledge, only one intervention has been identified that aims to reduce IPVAW public stigma among professionals. This was an educational intervention conducted by Mason et al. (2017) with the objective of reducing stigmatizing beliefs and attitudes toward survivors who also suffered from mental health and substance use problems. It is essential to emphasize the objective of reducing stigmatizing myths and attitudes, as other training interventions aimed at enhancing professional care in cases of VAW do not address this directly (e.g., Lathan et al., 2021). For this reason, interventions specifically aimed at reducing public stigma are important. They will not only seek to increase knowledge about IPVAW and protocols or ways to act but also to raise awareness about and decrease one’s own stigmatizing attitudes and behaviors.

Sereno et al. (2024) conducted a systematic review of studies conducted in the United States that analyzed the effectiveness of training programs designed to reduce biases (negative cognitive, attitudinal, and/or behavioral biases) related to IPVAW among professionals. The studies analyzed, including that of Mason et al. (2017), lacked sufficient detail regarding the training content, when available. Furthermore, the studies did not assess which components were involved in effectiveness. These findings make it challenging to determine the precise actions required to reduce public stigma. Moreover, the evidence did not support the assertion that experiential or long-duration interventions are more effective, although some studies have claimed otherwise (e.g., Lee et al., 2021). As Sereno et al. (2024) suggested, this should be further investigated.

In addition to findings from the United States, it is worth highlighting a number of very recent interventions aimed at professionals and designed to improve care in cases of VAW. These findings provide valuable insights into how interventions should be designed. First, it is recommended that training include content on VAW, the role of professionals in responding to cases of VAW, appropriate ways to approach these cases, trauma and crisis situations, cultural and legal influences, and resources available at the international, national and regional levels. It is also important to provide training in professional skills such as those outlined in the LIVES framework: Listen with empathy, Inquire about needs and concerns, Validate, Enhance safety, and Support and/or facilitate social support (Arora et al., 2021, 2023; Lee et al., 2021; Sakellari et al., 2024).

The literature recommends the use of participatory methods, such as role-playing (Arora et al., 2021, 2023; Lee et al., 2021; Sakellari et al., 2024). Also, the promotion of experiential and interdisciplinary learning (Sakellari et al., 2024). In implementing the intervention, a capacity-building approach with cascade training is recommended, where training is delivered by professionals who are experts in VAW and activist women (Arora et al., 2021, 2023), including IPVAW survivors (Lee et al., 2021; Sakellari et al., 2024).

Finally, as previously referenced, workplaces also serve as sites where gender biases and exclusionary practices are reproduced (Acker, 1990; Connell, 2009). Consequently, it is imperative to acknowledge that training professionals alone is not sufficient to bring about a change in attitudes. Rather, it must be accompanied by organizational changes to create an ecosystem supportive of adequate IPVAW responses (with clear regulations, mandatory training provisions, the reward of appropriate interventions by professionals, etc.) (Arora et al., 2021, 2023; Lin et al., 2021; Wu et al., 2020).

### The Current Study

In Spain, where this study was conducted, the incidence of physical and/or sexual IPVAW among women over the age of 15 is 14.2% (Government Office against Gender-Based Violence [GOGBV], 2019). This figure is relatively low compared to that in other European Union countries (European Union agency for Fundamental Rights, 2014) and globally (WHO, 2021). The literature indicates that this could be attributed to the preventive and educational initiatives implemented with the approval of Organic Law 1/2004 on December 28 (BOE, 2004). This legislation is regarded as one of the most advanced internationally in terms of prevention and protection against IPVAW.

In 2017, a State Pact was ratified that enhanced the previous legislation, aligning it with the standards set forth by the Istanbul Convention for the prevention and eradication of violence against women. The Pact required that the training of professionals be mandatory, standardized, and evaluable and that the institutions involved in the support system work in a joint and coordinated manner. Additionally, it promoted that survivors have access to professional assistance without the need to file a complaint.

Despite the above, a social movement has emerged that resists previous advances, often subtly and in the name of democracy (Calderón, 2020). With regard to professionals, recent national studies (e.g., Pastor-Gosálbez et al., 2021) warn of the secondary victimization of survivors. Also, of the necessity to continue training professionals in VAW and equality.

As observed by Yang et al. (2021), the existence of a legal framework that does not legitimize IPVAW is not incompatible with the existence of cultural norms that continue to contribute to its legitimization. Nevertheless, it must be acknowledged that Spain is a country that could be classified as privileged in terms of progress in the fight against IPVAW. Indeed, within Europe, Spain stands out as a country with an extensive body of research on myths and negative attitudes toward IPVAW and survivors (Gracia et al., 2020). Nevertheless, further work is required to enhance the services provided by professionals, who are not exempt from stigma (Durán-Martínez et al., 2022; Pastor-Gosálbez et al., 2021).

The research gaps related to decreasing professionals’ stigma against IPVAW survivors are, first, the lack of interventions that address both cognitive and behavioral biases (Sereno et al., 2024). It is often the case that interventions result in changes in attitude or beliefs, yet they fail to assess whether behavior also changes (e.g., Lee et al., 2021) or, when they do, observe that it does not (e.g., Mason et al., 2017; McMahon-Howard et al., 2013). Second, achieving long-term attitude change remains a challenge (Arora et al., 2021; Campbell et al., 2020). Third, there is a paucity of studies (e.g., Crowe & Murray, 2015; Mason et al., 2017) that focus on the various professionals involved in integrated, multidisciplinary care.

The challenges outlined thus far prompted the decision to conduct an interview-based study with professionals working in Spain in various sectors involved in the integrated care of survivors. The objective was to analyze the actions they considered relevant to preventing and reducing the stigma that still exists among professionals. Given that they work in a privileged legislative environment (Spain), these recommendations can serve as a reference for improving professional practice at the international level.

This project analyzed the international literature on public stigmatization of IPVAW over the last decade, the functioning of public stigmatization exercised by professionals in Spain and its consequences for survivors. These findings have been published elsewhere (Murvartian et al., 2023a, 2023b, 2024a, 2024b).

## Method

A critical feminist social constructivist epistemological approach (Wigginton & Lafrance, 2019) was adopted. From this perspective, it is assumed that there is no single interpretation of the object of study; rather, it is constructed by the researchers in the specific context of the investigation. In this sense, the interpretation was determined by the authors’ position, which was informed by the understanding of IPVAW according to the feminist perspective (Barnett et al., 2016; Dobash & Dobash, 1979), the recognition of survivors’ agency (Barrios et al., 2021), and the understanding of stigma from an intersectional perspective (Cárdenas, 2023; Crenshaw, 1991).

### Participants

The first author (research leader; RL) was responsible for recruiting participants. Purposive sampling was used to include somewhat diverse participants (e.g., different professions, years in practice) working in law enforcement, legal, health, psychological and social intervention. The RL distributed a flyer soliciting volunteers through professional networks nationwide and through recommendations of eligible participants from known professionals who work with survivors. The number of participants increased as data were analyzed to deepen the analysis and achieve the study’s objectives. Recruitment of new participants ceased when no new categories, relationships between categories, or participant perspectives emerged (data saturation, Rohleder & Lyons, 2014). Dworkin (2024) emphasizes that saturation is reached when collecting additional data no longer produces new relevant information. In her updated review, Dworkin notes that while she originally recommended 25 to 30 interviews in 2012, more recent studies have found that saturation can sometimes be achieved with 9 to 17 interviews, particularly in homogeneous populations and when research objectives are clearly defined. In our study, data saturation was reached with 25 professionals, aligning with Dworkin’s recommendations and exceeding commonly reported sample sizes in the literature (Hennink & Kaiser, 2022). No financial compensation was provided to the participants.

The participants (*n* = 15 women; *n* = 10 men) ranged in age from 26 to 65 years (*M* = 43.68; SD = 12.47). Years of practice ranged from 1.5 to 41 years. All the participants worked in one of the four aforementioned professional sectors and had a variety of professions. The participants worked in different regions of Spain, with only two employed in rural areas and the remainder in urban areas. Thirteen participants worked in specialized IPVAW positions, where they assisted survivors on a daily basis. The remaining professionals assisted survivors less frequently. However, all participants had received IPVAW training. For further details regarding the participants, see Table 1, in which they are identified by fictitious names.

**Table 1:** Characteristics of the participants

Sector	Name	Age	Gender	Profession	IPVAW-specialized services	Years in practice
Healthcare	Dolores	63	W	Physician	No	35
	Andrea	30	M	Physician	No	3
	Lindsay	49	W	Nurse	Yes	26
	Gabrielle	24	W	Nurse	No	2
	Manuel	56	M	Paramedic	Yes	26
Psychological intervention professionals	Charles	30	M	Private practice therapist	No	2
	Hanna	28	W	Private practice therapist	No	2
Social services	Katarina	56	W	Support counselor at a Municipal Center for Women’s Information	Yes	28
	Rosa	51	W	Family therapist	No	26
	Leo	58	M	Support counselor at a Regional Center for Women’s Support	Yes	25
Social intervention professionals	Giulia	65	W	Social educator in a Municipal Center for Women’s Information	Yes	41
	Rocío	31	W	Social worker in social services	No	11
	Jennifer	33	W	Social worker in social services	No	2
Association	Michael	37	M	Social worker in an association	No	13
Association and social services	Peter	49	M	Social worker in an association and in social services	Yes	15
	Pablo	30	M	Social worker in an association and in social services	Yes	6
Law enforcement	Esther	45	W	Local police officer	Yes	2
National police	Judith	26	W	National police officer	No	1.5
	Richard	45	M	National police officer working in Family and Women’s Services	Yes	18
Legal professionals	Fernando	35	M	Defense attorney	No	12
	Betty	48	W	Lawyer	No	21
	Alexandra	47	W	Legal advisor at a Municipal Center for Women’s Information	Yes	17
	Sarah	60	W	Legal advisor at a Municipal Center for Women’s Information	Yes	30
	Robert	43	M	Court clerk at the mixed gender-based violence court	Yes	8
	Martin	53	M	Defense attorney for the survivor	Yes	23

M, man; W, woman

### Procedure and Measures

The RL was responsible for the data collection. First, informed consent was obtained from the professionals. Subsequently, individual semistructured in-depth interviews were conducted (see Appendix), the script of which had been developed by the research team of the project in which this work is framed, audited by a group of experts, and piloted with three professionals before the project commenced. The aim of the interviews was to explore the functioning of professionals’ public stigma toward survivors and the consequences of this stigma, and to identify recommendations for combating stigma. Question 17 explicitly addressed the object of the present study, which constitutes the majority of the data. However, when relevant excerpts from responses to other questions were identified, they were also analyzed. The duration of the interviews ranged from 45 to 90 min. The candidates were given the option of being interviewed in person or via video call. Only one opted for an in-person interview; the remainder chose to be interviewed via video call. Once the interviews concluded, sociodemographic data were collected. The interviews were audio-recorded and transcribed verbatim.

### Data Analysis

The RL was responsible for conducting the data analysis, which consisted of a thematic analysis (Braun & Clarke, 2019). This process entailed the generation of a series of codes for relevant excerpts from the interviews. The codes were then organized into broader themes and subthemes through an iterative process. This involved the creation of new themes when the data did not fit into the existing scheme or the redefinition or combination of existing themes and subthemes. This process was repeated until no further new information relevant to the objective was identified. Once the final scheme of themes and subthemes was obtained, the definitions of these and the relationships between them were further elucidated. No data analysis software was used.

To ensure the confirmability of the data, the RL maintained a reflective attitude on how her position could influence her interpretations. She took notes on her results, revisiting them repeatedly to define and redefine categories in her analyses, eliminating some, adding new ones, or combining them. She also took notes on the challenges encountered and met with the second author to discuss them and reach a broader and novel perspective on the phenomenon under study. Additionally, to enhance the confirmability of the data, one third of the interviews were transcribed and analyzed independently by both authors, a proportion deemed appropriate by the literature. Subsequently, the two authors met and discussed the analyses together, once more debating those aspects that had raised doubts or on which they had disagreed.

Once the final scheme of themes and subthemes was obtained, the member checking technique was employed to enhance the credibility of the results (Braun & Clarke, 2013). A meeting was held with the participants, during which the results were presented, and the participants were invited to make suggestions regarding these results. The suggestions collected were subsequently incorporated into the final results.

## Results

The recommendations suggested by the participants to combat professional’s public stigma were divided into two main themes: actions to prevent stigma in society and actions to prevent stigma among professionals. The latter were further divided into individual-level actions and structural-level actions. The following paragraphs will elucidate the aforementioned supracategories and subthemes found within each one, accompanied by representative excerpts from the interviews. Table 2 presents the themes and subthemes in a schematic format.

**Table 2:** Scheme of themes and subthemes of actions to combat professionals’ public stigma against intimate partner violence against women survivors

Actions to prevent stigma in society	Actions to prevent stigma among professionals	
	Structural-level actions	Individual-level actions
Promoting gender equality education from an early age, a matter for everyone	Providing institutions with resources and protocols that would allow for IPVAW-specialized assistance	Maintaining a reflective attitude and referring to another colleague when necessary
Ensuring proper and ethical handling of information on IPVAW cases by the media	Ensuring competent professionals	^a^Providing survivor-centered care based on empowerment
Raising awareness on IPVAW and gender equality among politicians	Evaluating the real effectiveness of psychosocial intervention programs targeting survivors	

Major categories (supracategories or themes) are marked in bold to distinguish them from subthemes

^a^Subtheme that emerged during member checking

### Actions to Prevent Stigma in Society

According to the participants, some of the reasons for professionals’ public stigma stem from societal factors to which they are exposed, to varying degrees, simply because they are part of society. One such factor is patriarchal values that we are all socialized into. In other words, professionals are not immune to perpetuating public stigma. Consequently, when asked how to combat stigmatization by professionals, participants emphasized the need for interventions targeting not only professionals but society as a whole. Katarina (woman, 56 years old, support counselor at a Municipal Center for Women’s Information, IPVAW expert) expressed: “This is an issue that is a matter for society as a whole. This is why it needs to be addressed at all levels. If we only focus on the legal system or the police, it won’t work. It has to be society-wide.”

Among these recommendations, the most frequently mentioned was promoting gender equality education from an early age, a matter for everyone. In other words, gender-equal socialization. Manuel (man, 56 years old, paramedic, IPVAW expert) explained: “There is a lot of work to be done with the whole of society. We are born without prejudices. If everything since birth were focused on addressing equality—in television broadcasts, in games, in education—future generations would be different, including professionals.” They emphasized that equality education should be considered a matter for everyone, not a battle between men and women or a problem linked to political ideologies. In line with this, they also proposed the implementation of a mandatory training curriculum against IPVAW in schools at the state level, independent of the political party in power. Furthermore, they underscored the significance of sensitizing teachers, as they serve as fundamental role models.

Second, they recommended ensuring proper and ethical handling of information on IPVAW cases by the media. This would entail presenting information in an objective, less sensationalist manner, refraining from contributing to the stereotypes and myths that exist in this regard. In the words of Sarah (woman, 60 years old, legal advisor at a Municipal Center for Women’s Information, IPVAW expert):

A woman is murdered. Is the news given in an objective way, or do we create a tabloid news story? We always fall into the same clichés, asking the neighbors. The neighbors haven’t seen anything because, in most cases, the abuser from the outside is a great guy. So, the neighbors often say, “Well, I don’t know. He was a nice man. We didn’t notice anything.” We need to approach this differently, with more objectivity, raising awareness and establishing a code of ethics on how to report the news.

Finally, raising awareness on IPVAW and gender equality among politicians was proposed. This was considered a means of preventing politicians from minimizing the severity of or denying IPVAW and encouraging them to actively participate in the fight against it. Sarah explained:

There’s also a lack of awareness in the political sphere [she is talking about IPVAW and gender equality]. Politicians mess up a lot, not only at the highest levels but also at local levels–the politicians who are closest to us. Also, they leave the problem in the hands of gender equality offices, though this is a cross-cutting issue. You are also obliged to do some sort of activity in town planning…in whatever is related to the subject. Once they’ve got their gender equality offices up and running, they’re pretty happy.

### Actions to Prevent Stigma Among Professionals

In accordance with the analysis, a series of recommendations will be developed below with the specific objective of preventing public stigma among professionals. These guidelines aim to promote awareness and encourage reflective practice. Also, to foster a more supportive professional environment.

#### Structural-Level Actions

On the one hand, participants mentioned a set of actions targeting institutions and policies. These actions aim to support systemic changes that challenge the structural barriers sustaining gendered stigma. First, participants proposed providing institutions with resources and protocols that would allow for IPVAW-specialized assistance. Jennifer (woman, 33 years old, social worker in social services) stated: “It’s crucial to make sure there are resources and services available to support survivors in institutional settings. These resources should include economic, material, and human resources.” In line with this, developing protocols for intervening with IPVAW survivors that are understandable and accessible to all professionals was highlighted. This would assist professionals in providing high-quality care. The mere existence of protocols was deemed insufficient; efforts should be intensified to design them in a format that is easily understandable. Lindsay (women, 49 years old, nurse, IPVAW expert) specified: “It’s important to work with the right protocols and to make sure everything is clear. One of the things that was already introduced in the 2018 protocol was to put some quick guides—some algorithms—so that professionals can quickly and visually see what they have to do.”

Among structural-level actions, the most frequently mentioned ones had to do with ensuring competent professionals. This means professionals who are aware of IPVAW, who have received proper training, who are cooperative, and who know how to link survivors to other services so that they can receive adequate and continuous care. Recruiting personnel with a genuine awareness of IPVAW was identified as the initial step in this process. In addition to confirming that professionals had received training on the subject, participants believed that it was necessary to ascertain the degree of awareness during the selection process. Martin (man, 53 years old, defense attorney for the survivor, IPVAW expert) stated:

First, we need to select candidates. You might have a lot of training, but I want to know what you think of IPVAW and if you’re aware of it. Not everyone is right for the job. Also, take a look at their resume and any experience they’ve had with solidarity work or supporting marginalized groups. This shows they’re aware of and committed to the rights of minorities.

To ensure competent professionals, many participants also recommended providing adequate training for professionals. According to the interviewees, this training should be mandatory, continuous, scientific, updated, and properly evaluated. It should also include theoretical content on IPVAW, what IPVAW recovery consists of, and how to adequately intervene in cases of IPVAW. Furthermore, they asserted that training should be interdisciplinary, incorporating psychosocial content for law enforcement and legal professionals. This was considered crucial for understanding survivors (the challenges they face, the decisions they make, etc.) and for avoiding judging or blaming them. Judith (woman, 26 years old, national police officer) explained:

Training is the key. Having studied psychology and a bit about these issues, I’ve learned that the police officer on the street doesn’t get much training on IPVAW. They don’t know how to act or why IPVAW happens. In specialized IPVAW units there’s a little more training, but it’s not updated or of psychological nature. About knowing at least how to take a report...well, there’s not much training either.

Another observation was that training should not only include theoretical content but also involve the active participation of professionals and include an experiential component that would facilitate awareness of their own misconceptions and negative attitudes toward IPVAW, survivors, and women in general. This would help to transform beliefs and attitudes that are often implicit and hinder understanding of women. In the words of Katarina (woman, 56 years old, support counselor at a Municipal Center for Women’s Information, IPVAW expert):

Training for professionals, but the right training. A twenty-hour course on the Cycle of Violence isn’t enough. It has to transform the professional and “cleanse the professional of patriarchy.” It has to start with me, with what I’ve experienced and how I’m still influenced by patriarchy. Then I can learn the theoretical content (how IPVAW works). You can know how IPVAW works, but if you haven’t “cleansed yourself of patriarchy,” you might be perpetuating it.

Additionally, it was proposed that training should facilitate self-analysis, enabling professionals to become aware of their own stigmatizing reactions during their practice. Charles (man, 30 years, private practice therapist) stated:

We need to host workshops where counselors look in the mirror and examine how they perpetuate IPVAW, victimize women, and burden them with the responsibility to fix all of it. They don’t realize that women are here to get our help in dealing with this.

In addition, participants suggested that the training should include the voices of survivors, with the possibility of survivors themselves facilitating the training. It was emphasized that survivors should narrate the stories of stigmatization they had encountered during their interactions with various support services, as this would be more impactful and contribute to stigma awareness. Andrea (man, 30 years old, a physician) explained:

[Talking about training] It’s crucial to get to know survivors and have them share their stories with professionals. It’s important for providers to understand how survivors felt and what happened. Some healthcare professionals might have told them, “No, you’re lying to me.” “I lied on Monday and on Sunday I had my jaw and teeth knocked out because I got beaten up…and I was lying, right?” [Imitates a survivor]. These things affect the staff because they’re based in reality.

Another of the proposed actions to ensure competent professionals was promoting and providing resources for the existence of spaces for supervision and teamwork from a horizontal, collaborative perspective that incorporates affection. On the one hand, teamwork and supervision of one’s own practice by others facilitates self-reflection and the prevention of stigma, since what one does not realize, the other person does. Hanna (woman, 28 years old, private practice therapist) explained: “It’s important to have supervision from other professionals. Working as a team helps to avoid that kind of prejudice and to have a colleague tell you, ‘Hey, look at what you’re thinking about this. Be careful. Don’t stigmatize. Don’t be prejudicial’.” On the other hand, it was proposed that teamwork incorporated affection, respect, and the valuing of colleagues and that it was collaborative and horizontal. It was noted that to eliminate the verticality that often characterizes professional care and stigma in general, it was necessary to begin doing so among peers. This was expressed by Giulia (woman, 65 years, social educator in a Municipal Center for Women’s Information, IPVAW expert):

There’s a lack of spaces for dialogue, reflection, analysis, and for sitting down together and just talking: “Hey, what do you think about this?” It’s important to know each other’s visions, what kind of support we can be for each other at a given moment, how we can connect with survivors, and to recognize when we don’t connect so that another partner can step in. That’s how we can work on being humble and showing affection. Affection means treating the person with respect and dignity. We need to learn to recognize emotions, share when we are feeling helpless, share resources and deficiencies with our colleagues...because we have them in common.

It was recommended that collaborative work be extended to professionals from other services, with the aim of continuing the help provided or offering another type of help that could not be provided from a particular service. In this sense, it was indicated that facilitating that professionals know and have access to available services with which to connect survivors is also a priority. In the words of Lindsay (woman, 49 years, nurse, IPVAW expert): “I know that this is as far as I can go, that I can take care of the survivor initially, but I need to give her continuity, so I need to have the tools to be able to refer her to someone who can take care of her further.” The necessity for these services to be truly accessible was highlighted. Martin (man, 53 years, defense attorney for the survivor, IPVAW expert) explained: “We need to be provided with a network of resources, but not web pages or…I mean names and surnames, phone numbers that are available when survivors really need them.”

Finally, at a structural level, some participants agreed that evaluating the real effectiveness of psychosocial intervention programs targeting survivors was imperative. This evaluation should not be limited to the number of women who participated or attended the programs regularly, which is the simplest thing to do. Rather, it should consider other elements inherent to the complexity of IPVAW recovery. Peter (man, 49 years old, social worker in an association and in social services) explained: “Programs are often evaluated based on numbers. It’s important to remember that survivors face real challenges to get services and to attend workshops. It’s a slow process.” During the course of the interview, Peter explained that professionals lacked an understanding of survivors’ lack of attendance at the workshops. According to Peter, this was because they were not aware of the difficulties survivors faced during recovery, which reinforced public stigma. The fact that the effectiveness of the programs is evaluated by counting attendance at the intervention sessions contributes to the fact that professionals only pay attention to this.

#### Individual-Level Actions

Participants also identified a number of actions that professionals should implement in their practice to prevent public stigmatization of survivors. The most frequently mentioned actions involved maintaining a reflective attitude and referring to another colleague when necessary. This entailed remaining attentive to one’s own stigma that may emerge in professional practice. According to Leo (man, 58 years old, support counselor at a Regional Center for Women’s Support, IPVAW expert), professionals should be attentive to their own thoughts and feelings when faced with the decisions or reactions of survivors. This is the first step in becoming aware of and questioning the stigmatizing social beliefs they have learned throughout their lives and the associated prejudices or stigmatizing behaviors. This process could help professionals remedy, if not eliminate, their own stigma.

Some women will attack you if you don’t give them what they want to hear or...and you feel the attack. You don’t want to acknowledge it because, well, poor thing, on top of what she has been through...The problem is that you’re reacting without realizing it, so you start to physically move away and change your facial expression. You have to be able to recognize that you’re feeling attacked so that you can act differently. You can say, “Let’s stop the session here, let’s do things differently.” The same should be done with stigma. “This woman, what she’s telling me, I’m not believing it. Wait, let’s stop”, then you can start doing something different. It’s an exercise of introspection; being able to recognize and, if not eliminate that stigma, at least ensure it doesn’t cause harm.

Participants advised that professionals begin practicing this introspective exercise in other areas of their lives as well. According to Richard (man, 45 years old, national police officer working in Family and Women’s Services, IPVAW expert): “We can’t just stick to the protocols. We have to get involved, take a look around, and ask ourselves: Am I being fair with my wife, with my sister? If we would just take a moment to look inward…”

Once this introspective process is completed, participants recommended considering referral to a colleague if professionals do not feel capable of providing stigma-free service. Charles (man, 30 years old, private practice therapist) explained: “If you think you might do more harm than good, you should act accordingly. If I don’t feel comfortable, you should refer the survivor to another professional who knows how to act professionally and move forward with the intervention.”

In conclusion, maintaining a reflective attitude involves professionals developing the ability to recognize and challenge their own potential biases or stigmatizing thoughts and reactions when working with survivors. It begins with self-awareness: professionals must actively monitor their feelings, thoughts and behaviors, especially when they notice discomfort or skepticism in response to a survivor’s words or actions. This introspection is crucial because it allows professionals to recognize when they may be unconsciously holding biased views, such as disbelief or judgment. Once these reactions are recognized, they can be addressed by either stepping back, rethinking their approach, or seeking support from colleagues to ensure that their responses do not negatively impact the survivor. In essence, being reflective is about creating a space for self-examination to prevent personal biases from influencing the professional relationship and ultimately ensuring that the care provided remains compassionate, unbiased, and effective.

The remaining proposed actions focused on providing survivor-centered care based on empowerment. This approach emphasizes placing survivors at the center of their recovery, ensuring they are actively involved in decisions, and fostering responsibility and autonomy. First, participants emphasized the importance of maintaining close contact with survivors and paying attention to the particularities of each IPVAW case. Being in contact with survivors and listening to their unique stories would help to reduce stigma, including stereotypes about survivors (e.g., stereotypes about survivors, lack of attention to each woman’s individual needs, etc.). Rocío (woman, 31 years old, social worker in social services) explained: “You need to work closely with these women and focus on their individual needs. It seems like survivors are just numbers. All that matters is the statistics, but when you sit down with a survivor, all that goes out the window.”

Participants were critical of the top-down approach in survivor care. They highlighted how women’s agency is often overlooked, with decisions being made on their behalf rather than empowering them. The need for an empowerment-based approach was also stressed. Giulia (woman, 65 years old, social worker, IPVAW expert) expressed:

The empowerment of women must also be reflected in small actions. For example, I accompany a woman to do the relevant bureaucratic procedures, and I always stand behind her and I let her speak. I try to give them hope that recovery and liberation from violence are possible, and that they must be the protagonists of their own change. It is about establishing an agreement in which, with the support of the professional, they take responsibility for achieving it.

Building on the need for survivor-centered care, participants also noted that professionals often prioritize the well-being of minors, sometimes at the expense of intervening in the abusive situations affecting their mothers. This has led to cases where children are removed from their homes. In this regard, professionals emphasized the importance of prioritizing the recovery of survivors while also ensuring that minors are protected. Rosa (woman, 51 years old, family therapist) stated: “Taking care of children shouldn’t mean overlooking the safety and recovery of their mothers. It’s crucial to adopt a more holistic approach, one that supports mothers and protects children without making separation the first option.” Although this suggestion could be classified as a structural-level action, it was considered an individual-level action because professionals can implement it when assessing the situation of minors and the family and deciding on the appropriate course of action.

## Discussion

As previously indicated, the objective of this study was to analyze the actions deemed relevant by a number of professionals in order to prevent and reduce professionals’ public stigma toward IPVAW survivors. The results were organized into major themes or supracategories. To facilitate reading, the discussion follows this structure.

### Actions to Prevent Stigma in Society

It was emphasized that society must be educated in equality from an early age and in a cross-cutting manner, through content shown on television, in schools, etc., as suggested by the literature (MacGregor et al., 2017; Murray et al., 2016; Smye et al., 2020). Although progress in recent decades is undeniable, it is imperative to make psychological intimate partner violence, primarily controlling behaviors (Meil, 2014), and intimate partner sexual violence more visible, as they remain the most normalized forms of violence and therefore the most difficult to detect (Ministry of Health, Social Services and Equality, 2012; Murray & Crowe, 2017). It is crucial to highlight the importance of education at the university level, particularly in disciplines such as nursing, psychology, and law, which prepare future professionals (Murvartian et al., 2024b; Sakellari et al., 2024). This approach has been successfully implemented in certain countries (e.g., Turkey, Toplu-Demirtaş & Aracı-İyiaydın, 2023), and its effectiveness in Spain has been a subject of debate (e.g., Freijomil-Vázquez et al., 2022; Gómez-Fernández et al., 2017).

Furthermore, education should also be carried out through the media. The Spanish media has played a pivotal role in raising social awareness and advancing public policies on IPVAW (Cuesta-Ramírez, 2021). Nevertheless, it is necessary to persist in advocating for the ethical management of information on IPVAW. Despite the fact that there have long been recommendations from the GOGBV for this purpose in Spain (Cuesta-Ramírez, 2021), ethical management can be complex given the politicization of the media (Guerrero-Solé, 2022), since IPVAW is denied from certain political sectors (Cabezas et al., 2023; Varela-Guinot, 2021). In alignment with this, the proposal was made to raise awareness among political leaders. Although politics is a reflection of public opinion and denialist discourses existed prior to the formation of IPVAW negationist political parties (Cabezas et al., 2023), stigmatizing attitudes are now publicly legitimized by politics (Rinken, 2019).

According to the literature, it is imperative that institutions and policies continue to intensify their efforts to make IPVAW and support resources more visible, educate, and raise awareness (Murray & Crowe, 2017; Murray et al., 2016; Smye et al., 2020). Structures can facilitate the denormalization of IPVAW and its recognition as a public problem, which in turn can prevent stigma (Barnett et al., 2016; Pescosólido & Martín, 2015). Murray and Crowe (2017) additionally observed that it is crucial for professionals, given their expertise, to engage in the implementation of the recommendations previously outlined.

### Actions to Prevent Stigma Among Professionals

In addition, recommendations directed at professionals were identified, which could be at the structural or individual level. Some focus on systemic changes, while others encourage personal reflection and professional development. Both approaches aim to reduce stigma and improve practice.

#### Structural-Level Actions

The most frequent recommendation was to ensure competent professionals, mainly through appropriate training. As evidenced by the literature (e.g., Almqvist et al., 2018; Sakellari et al., 2024), the necessity of training professionals in appropriate responses to IPVAW cases has been widely emphasized. This training should extend to those working in specialized care services for survivors, who are not always adequately trained despite the creation of specialized positions mandated by law (Durán-Martín et al., 2022; Maldonado, 2020).

The importance of providing institutions with resources and protocols that would allow for IPVAW-specialized assistance was also highlighted by participants. However, while the availability of accessible and understandable protocols for intervention and resources is undoubtedly necessary, it is not sufficient. For example, those professionals who apply the protocol the least have themselves highlighted their lack of training as the main underlying reason (Valdés Sánchez et al., 2016). Additionally, they caution that the protocols are too general and that what is really needed is for them to be more specific and adapted to the demands of their daily work (Grant, 2014). Murray and Crowe (2017) additionally propose that professionals be trained in a variety of intervention approaches, thereby enabling them to select an appropriate course of action for each survivor.

It is also imperative that institutions provide sufficient resources to ensure the delivery of adequate care (Cárdenas et al., 2024; Murray & Crowe, 2017). However, this is insufficient; it is also necessary to facilitate professionals’ contact with other services and coordinated teamwork (Murray & Crowe, 2017). To this end, Murray and Crowe (2017) proposed that each service provides training to familiarize others with their methods of operation, thereby enabling all professionals to understand the functioning of other sectors. Furthermore, they propose that when multiple services are providing assistance to the same survivor, they should enter into coordinated working arrangements and share case information. This approach not only permits the provision of care that is tailored to the specific needs of women but also facilitates the continuity of care over time. Moreover, it helps to mitigate some of the challenges associated with resource limitations.

Going back to the importance of training, which was very frequently mentioned, it is worth mentioning that professionals have expressed frustration with their inability to provide appropriate care (Cubells & Calsamiglia, 2018; Portela-Romero et al., 2020), which increases the risk of engaging in stigmatizing behaviors. To ensure the effectiveness of the training, based on the literature and the most novel and/or relevant suggestions from participants, the content should align with what was stated in the introduction (Arora et al., 2021, 2023; Lee et al., 2021; Sakellari et al., 2024). Above all, training should facilitate a thorough understanding of IPVAW functioning and the IPVAW recovery process (García-Jiménez et al., 2020). This encompasses an understanding of the micro- and macrolevel barriers that women face, which must be considered from an intersectional perspective (Barrios et al., 2021; Cárdenas, 2023; Pokharel et al., 2020) and in a culturally sensitive manner (Cárdenas et al., 2024). Furthermore, it is essential to acknowledge that women consistently take action to combat violence in a context where their degree of control over their lives varies considerably from situation to situation (Black et al., 2020).

Incorporating survivors’ voices in training represents a promising strategy for achieving the above-mentioned objectives (Gómez-Fernández et al., 2017; Lee et al., 2021; Murray & Crowe, 2017; Murray et al., 2016; Sakellari et al., 2024; Smye et al., 2020) and recognizing that each experience of battering is unique (Barrios et al., 2021). Interestingly, none of the interviewees mentioned the potential integration of survivors into professional teams as a form of mutual support. While the implementation of such strategies in Spain remains limited at both organizational and legal levels, their absence from the participants’ reflections is noteworthy. In the field of mental health, the inclusion of peers with lived experience is not uncommon and has been shown to be effective in promoting recovery (Smit et al., 2023). Although still in its early stages, some initiatives in Spain are beginning to move in this direction. With regard to the implementation of the training, the approach of capacity building with cascade training, as proposed by Arora et al. (2021, 2023), could be a good strategy.

Another key aspect is that training for law enforcement and legal professionals should incorporate content of a psychosocial nature. Ward-Lasher et al. (2017) even proposed that social workers should be the ones to provide training. This is also applicable to the healthcare setting, where a biomedical approach focused on symptoms still predominates (Gómez-Fernández et al., 2017). Training should be interdisciplinary (Sakellari et al., 2024), as should the work.

One of the most significant challenges in the design of effective future training interventions is the need to address both stigmatizing beliefs and behaviors (Sereno et al., 2024) and to ensure that changes are sustained over time (Arora et al., 2021; Campbell et al., 2020). In this context, the interviewees proposed that training should be experiential, with the objective of enabling professionals to become aware of and reflect on their attitudes and behaviors, as well as on the roots of stigma (e.g., the patriarchal values they have internalized, their personal experiences with IPVAW, etc.). One illustrative example is the use of theater-based techniques, which permit the recreation of IPVAW scenarios in a manner that closely resembles reality. As is the case with many other creative practices, identity, experiences, beliefs, emotions, and ways of acting, more or less implicit, come to light. Moreover, they permit the practice of alternative, more positive ways of acting (Curioni et al., 2023; Heard et al., 2020), such as sensitive ways of communicating with survivors (European Institute for Gender Equality, 2020), which substitute for stigmatizing responses.

It is also recommended that participatory methods, which include the use of indirect and direct reflective questions and discussion, be employed (Arora et al., 2021, 2023; Lee et al., 2021; Sakellari et al., 2024). In the interviews conducted with professionals, it was observed that they encouraged reflection on stigma. Some even indicated that it was the first time they realized that they had exercised stigma (Murvartian et al., 2023a, 2024b).

In addition to training, it is worth emphasizing another way of ensuring competent professionals that was mentioned by participants: promoting a culture of horizontal teamwork, in which tasks of supervision of practice are carried out and affection is incorporated. This would help to combat the verticality (top-down approach) of the support system, which according to Serra (2023) permeates the assistance given to survivors. Moreover, the ability to share limitations and challenging experiences in these settings also facilitates the self-care of professionals, which is crucial for preventing the exercise of stigma (Kulkarni et al., 2015; Murray & Crowe, 2017).

#### Individual-Level Actions

A common recommendation proposed by the interviewees was that professionals should maintain an attitude of self-analysis regarding their own stigmatizing attitudes and behaviors (Álvarez et al., 2016). In this regard, Sabafrén and Vega (2004) posited that this awareness on the part of the professional serves as a “protective screen,” as it allows them to manage discomfort without compromising the quality of professional care. It allows them to do something about it (e.g., discuss it with other colleagues who can be of help, refer the case; Murray & Crowe, 2017).

The other suggestion was that it was necessary to work from a survivor-centered approach that was based on women’s empowerment. According to Álvarez et al. (2016), to support women survivors, professionals should adopt an empowerment approach that recognizes the fundamental role of patriarchal inequality in perpetuating violence. This approach focuses on enabling survivors to take control of their lives and choices, and promotes a shift from unequal power dynamics to egalitarian relationships. Empowerment is understood as a dynamic process through which people who have experienced marginalization develop the skills to critically examine and challenge the power structures that contribute to their subordinate position. In practice, this means more than simply building individual self-esteem and skills; it requires professionals to facilitate the development of awareness of gender power imbalances and to support the challenging of sexist beliefs. In addition, the focus should be on transforming relationships by promoting the survivor’s autonomy and ability to build equal relationships. Professionals can contribute to this by supporting survivors to understand the origins of inequalities, to identify their rights and strengths, and to work together to promote the changes necessary for them to become the architects of their own lives and to contribute to wider social change toward equality. This perspective sees power not as domination, but as the ability to control essential resources for development and influence. Ultimately, the goal is to cultivate autonomy and foster the development of relationships based on equality and mutual respect.

On the one hand, maintaining close contact with survivors and remaining attentive to their specific circumstances and needs was suggested. This not only helps to break down negative stereotypes, as explained by participants, but also helps to develop a protection plan. Ensuring that basic needs are met before encouraging survivors to report, to break the relationship, or before the professional reports the situation to the court is essential (MHSSE, 2012; Roush & Kurth, 2016).

On the other hand, professionals must situate women at the core of the intervention, providing them with guidance and support to regain control over their lives. In other words, the intervention must be tailored to their circumstances and the specific moment they are in, and the steps to be taken must be negotiated and agreed upon between the professional and the survivor (Álvarez et al., 2016). This approach counters paternalism and the blaming and abandonment of women by the support system, two poles that are part of the stigma (Murvartian et al., 2023a, 2024b).

It is imperative to respect women’s rhythms and refrain from coercing them to make decisions (Maldonado, 2020). Additionally, it is crucial to acknowledge and validate any progress, even if minimal (Murray et al., 2016). To empower them, it is also necessary to inform them of the potential barriers and risks involved in the actions that can be followed. In this manner, they are able to make informed decisions (Murray & Crowe, 2017), and the risk of retraumatization is avoided (González-Méndez & Hamby, 2021).

In relation to this empowerment-based intervention approach, González-Méndez and Hamby (2021) proposed a shift from a deficit model to a strengths model in which survivors’ personal strengths are promoted. Additionally, Alvarez et al. (2016) proposed that interventions should focus on fostering healthy relationships rather than on abuse. In this manner, paternalism is counteracted and serves to repair the harm caused by IPVAW through learning healthy relationship models.

Also linked to the empowerment of women, social intervention professionals were encouraged to address IPVAW before considering the removal of minors. It has been argued that women are often forced to choose between their relationship and their children (Maldonado, 2020). Instead, it is recommended that they be assisted in regaining the strength that they have lost due to abusive dynamics and in freeing themselves from violence, which is part of a commitment to prioritizing the well-being of minors (Murray & Crowe, 2017).

### Limitations

The main limitation of this investigation is that there may be valuable recommendations for the field of study that did not emerge from the interviews. An effort was made to ensure sufficient heterogeneity among participants to enhance the richness of the data and to reflect the diversity of professional sectors and professionals within each sector. Nevertheless, it was not feasible to access certain areas that could have potentially enhanced the data set, such as professionals employed in emergency centers and shelters. Future research similar to this study should also be conducted with professionals who were not represented.

### Conclusions

This study offers relevant recommendations to combat public stigma from professional helpers toward IPVAW survivors. At a general level, emphasis was placed on promoting gender equality education from an early age. Among the recommendations addressed specifically to professionals, which should be developed at the structural level, some were to provide institutions with resources and protocols to provide adequate care. Others were to ensure competent professionals through effective training and community workspaces to facilitate group discussions and supervision, among other actions. It is also important for professionals to feel valued by the institution and to provide resources for their care. Additionally, evaluating the real effectiveness of psychosocial intervention programs targeting survivors was suggested. At the individual level, professionals were advised to be reflective about their own stigmatizing attitudes and behaviors, which would enable them to refer cases to other professionals when appropriate or to take other actions to prevent stigmatizing responses. A survivor-centered intervention model was also advocated, which seeks to empower women and is based on the strengths of survivors and healthy relationships. This also implied that social intervention professionals should address IPVAW before considering the removal of minors.

## Data Availability

In view of the fact that the data are identifiable and of a sensitive nature, they are not deposited in a public repository. The data are available from the corresponding author.

## Appendix

### Interview Script

1. Could you please describe your current position, as well as how long you have been in this role?
2. Could you describe a significant instance in which you assisted an IPVAW survivor (the details of what occurred, your feelings, etc.)?
3. How do you typically feel at work when supporting survivors? Why do you feel this way?
4. What are the most challenging aspects of assisting survivors?
5. How do you typically address these challenges?
6. How would you define IPVAW?
7. Do you identify any common characteristics or circumstances among survivors?
8. In your experience, both as a professional and from life in general, what do survivors typically do in response to IPVAW?
9. What is your recommendation for how survivors should proceed?
10. Why do you believe survivors are sometimes reluctant to disclose the abuse, seek help, or pursue legal action?
11. The term “stigma” is often used in both the social sciences and colloquial language. Could you describe your understanding of the term “stigma”? (A brief definition is provided after the answer: “Let me provide a brief definition of this term, so that we can discuss it in the same terms throughout the interview.” Stigma is a social phenomenon that involves a set of erroneous beliefs, prejudices, and discriminatory behaviors by society toward certain people due to a particular condition or characteristic. For instance, individuals with mental illness or HIV are often the targets of stigma.
12. Do you believe that IPVAW survivors are stigmatized?
  - If the answer is yes:
    - 12.1.  Please describe the nature of the stigma.
  - If they are not sure:
    - 12.2.  Please indicate whether there are any other social conditions where you believe stigma exists.
      - If the answer is no, please proceed to question 19.
      - If the answer is yes:
        - 12.2.1.  What does that stigma look like?
        - 12.2.2.  Would you say something similar happens to IPVAW survivors?
          - If the answer is no, the interview ends.
          - If the answer is yes:
            - 12.2.2.1.  What form does it take?
  - If the answer is no, proceed to question 19.
13. Have you ever noticed other professionals in your field stigmatizing survivors?
  - If the answer is yes:
    - 13.1.  Please explain a bit and perhaps give an example.
  - If the answer is no, proceed to question 14.
14. Why do you believe survivors are stigmatized?
15. What impact do you believe this stigma has on survivors? Have you observed this firsthand in your professional or personal experience?
16. Do you think the stigmatization of survivors in society differs from that by professionals? Can you elaborate?
17. How do you think we can combat and reduce this stigma in professional settings when helping survivors?
18. Last, and to reflect on our own actions, do you think you may have ever contributed to this stigma at work? Can you elaborate?
19. Is there any additional information you would like to provide before we conclude?
